# Supplementary material for: Over-coupled resonator for broadband surface enhanced infrared absorption (SEIRA)
Source: Nat Commun. 2023 Aug 9;14:4814. doi: 10.1038/s41467-023-40511-7 (PMC10412556; doi:10.1038/s41467-023-40511-7)
Supplement: Supplementary file 1 — Supplementary information [file 41467_2023_40511_MOESM1_ESM.pdf]

Supplemental Material: Over-coupled resonator for broadband surface enhanced infrared absorption (SEIRA)

Laura Paggi,[1] Alice Fabas,[1] Hasnaa El Ouazzani,[1] Jean-Paul Hugonin,[2] Nikos Fayard,[2] Nathalie Bardou,[3] Christophe Dupuis,[3] Jean-Jacques Greffet,[2] and Patrick Bouchon[1,*]

[1]*DOTA, ONERA, Université Paris-Saclay, F-91123 Palaiseau - France*

[2]*Laboratoire Charles Fabry, Institut d'Optique Graduate School, CNRS, Université Paris-Saclay, 91127 Palaiseau, France*

[3]*Centre de Nanosciences et de Nanotechnologies (C2N), CNRS, Université Paris-Saclay, 10 Boulevard Thomas Gobert, 91120 Palaiseau, France*

* patrick.bouchon@onera.fr

1. ELECTROMAGNETIC DESIGN

1.1. Electromagnetic simulation and permittivity models

TABLE S1. Drude-Lorentz model of PMMA adapted from Tsuda et al.[S1]

n	$\omega_{0,n}(cm^{-1})$	A_n	$\gamma_{L,n}(cm^{-1})$	n	$\omega_{0,n}(cm^{-1})$	A_n	$\gamma_{L,n}(cm^{-1})$
1	752.25	3.18E-03	13.65	13	1361.50	1.09E-03	41.97
2	808.09	6.94E-04	15.51	14	1387.61	1.07E-03	15.80
3	825.19	1.13E-04	4.21	15	1434.77	1.34E-03	10.56
4	843.16	2.86E-03	23.09	16	1450.59	4.11E-03	25.15
5	913.82	1.68E-03	32.50	17	1481.89	2.12E-03	19.17
6	965.31	3.94E-03	26.70	18	1730.18	1.56E-02	19.40*
7	989.60	2.79E-03	14.68	19	2840.98	6.66E-05	15.32
8	1066.27	1.10E-03	13.56	20	2920.93	8.42E-04	60.94
9	1149.37	2.92E-02	31.12	21	2950.55	6.60E-04	18.80
10	1190.32	1.04E-02	22.12	22	2997.71	9.53E-04	36.68
11	1241.23	6.64E-03	21.38	23	3440.07	4.15E-05	33.89
12	1269.59	5.49E-03	24.66				

* modified value from original paper

Calculations were obtained using a B-Spline Modal Method and confirmed with the rigorous coupled wave analysis (RCWA) modal method [S2, S3]. The ZnS layer permittivity is modeled thanks to a modified Sellmeier model taken from *Klein et al.*[S4]. Chromium and titanium adhesion layers are modeled with Brendel–Bormann models from Rakic *et al.*[S5]. The gold layers are modeled using a Drude model and the PMMA permittivity is given by a Drude-Lorentz model adapted from Tsuda *et al.*[S1]. The Drude model for gold is given by the following equation:

$$\epsilon(\lambda) = 1 - \frac{1}{(\frac{\lambda_p}{\lambda} + i\gamma)\frac{\lambda_p}{\lambda}}, \tag{S1}$$

with $\lambda_p = 159\,\text{nm}$ and $\gamma = 0.0077$ to fit the literature data in the infrared [S6]. The Drude-Lorentz model for PMMA is given by the formula:

$$\epsilon(\omega) = \epsilon_\infty + \sum_n A_n \frac{\omega_{0,n}^2}{\omega_{0,n}^2 - \omega^2 + i\gamma_{L,n}\omega}, \tag{S2}$$

where $\epsilon_\infty = 2.162$ is the dielectric constant at high frequency, A_n is the amplitude of the nth oscillator, $\omega_{0,n}$ is its resonance frequency and $\gamma_{L,n}$ its damping factor.

1.2. Properties of the resonator

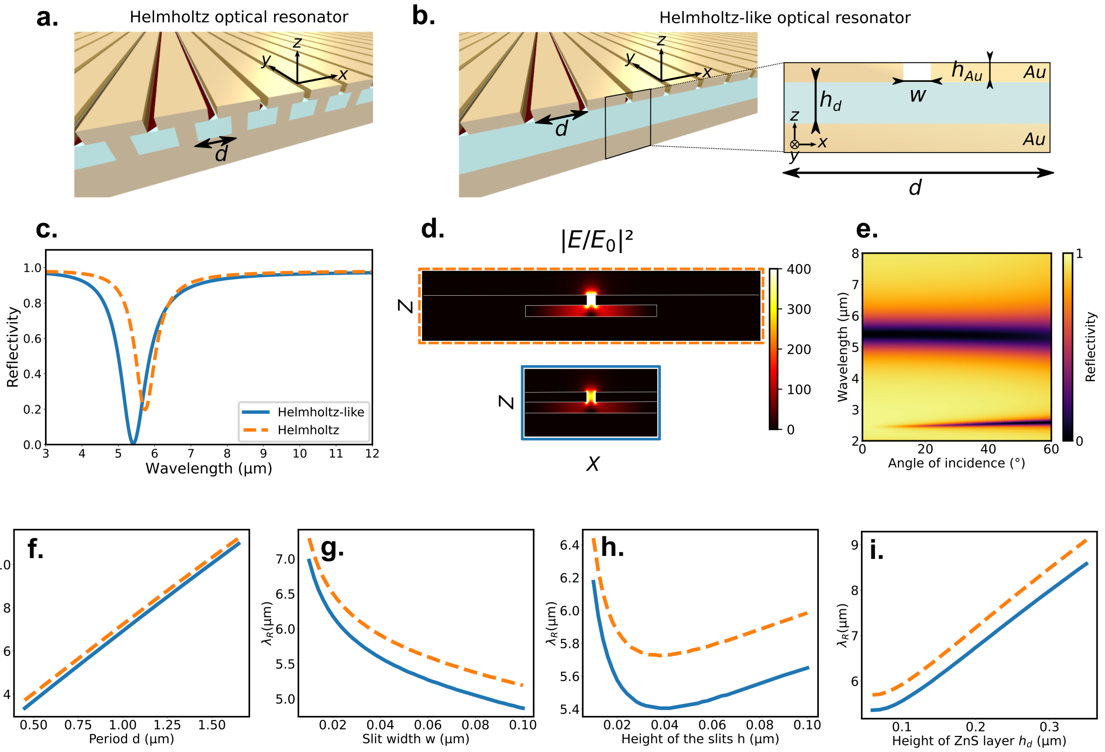

FIG. S1. **Helmholtz-like optical resonator.** **a** Helmholtz optical resonator as described in [S7], **b** Simplified design after removal of the dielectric cavities walls to form a continuous layer, **c** Reflectivity spectra of the Helmholtz resonator with a period of 2 µm (dashed orange line) and the simplified structure (blue continuous line). In both cases dimensions are $h_d = 50nm$, $h_{Au} = 45nm$, $w = 50nm$ an $d = 765nm$. **d** Normalized electric field intensity maps for both structures. **e** Angular stability of the simplified structure, **f-i** Evolution of the resonance wavelength as a function of the geometrical parameters for both structures.

Figure S1 shows the comparison of the optical Helmholtz resonator described by P. Chevalier *et al.*[S7] and the resonator investigated in the manuscript that is a simplified design.

Indeed, the dielectric cavities of the original design can be widened until complete disappearance of the metallic walls. The LC resonator description is still applicable as the slit concentrates the electric field and the cavity the magnetic field at resonance and the properties of the Helmholtz resonance are maintained, with a notable difference being the fact that the width of the cavity and the period are now identical.

1.3. Field enhancement

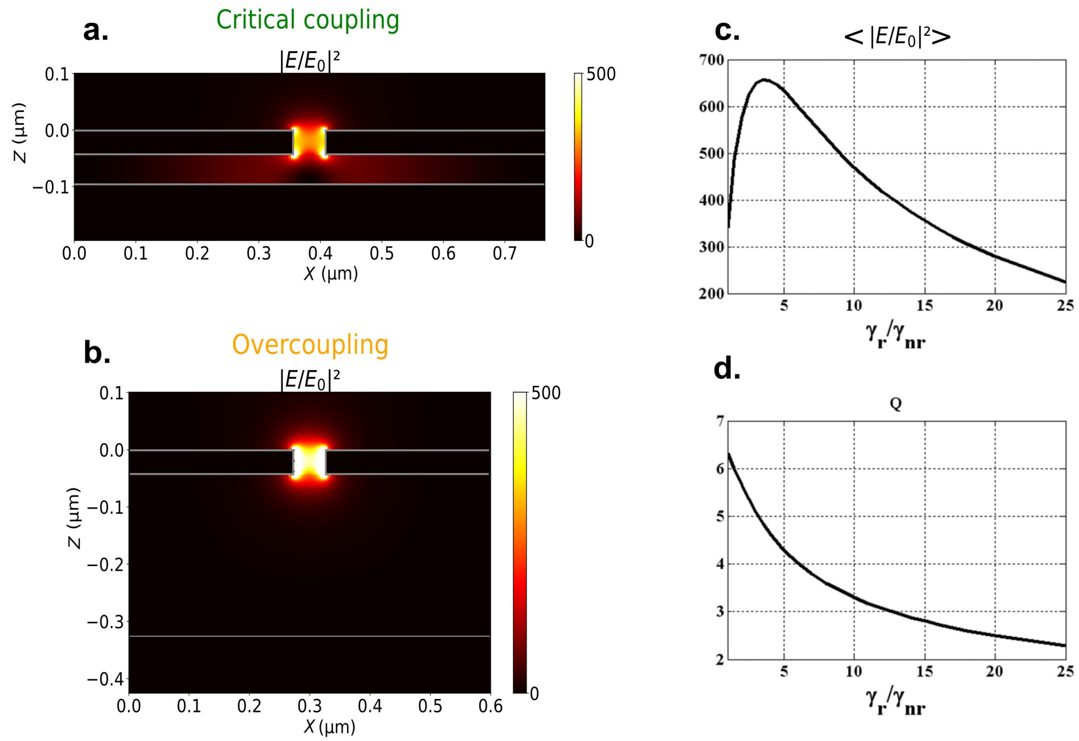

FIG. S2. **Electric field enhancement and coupling ratio.** **a** Normalized electric field maps of a critically coupled resonator with dimensions $h_{\mathrm{Au}} = 45\,\mathrm{nm}$, $h_{\mathrm{ZnS}} = 50\,\mathrm{nm}$, $w = 50\,\mathrm{nm}$ and $d = 765\,\mathrm{nm}$, **b** an over-coupled resonator with dimensions $h_{\mathrm{Au}} = 45\,\mathrm{nm}$, $h_{\mathrm{ZnS}} = 280\,\mathrm{nm}$, $w = 50\,\mathrm{nm}$ and $d = 600\,\mathrm{nm}$. **c** Evolution of the normalized electric field intensity avaraged on the slit section as a function of the coupling ratio $\gamma_{\mathrm{r}}/\gamma_{\mathrm{nr}}$ and **d** of the quality factor Q as a function of the coupling ratio γ_r/γ_{nr} calculated from resonators with constant slit dimensions $w = 50\,\mathrm{nm}$ and $h_{\mathrm{Au}} = 45\,\mathrm{nm}$ and constant wavelength of resonance of $\lambda_R = 5.78\,\mu\mathrm{m}$.

The field enhancement computed at resonance wavelength is plotted in Fig. S2a for a

resonator optimized near critical coupling ($\gamma_r = 1.18\gamma_{nr}$) and in Fig. S2b for a resonator in an over-coupled configuration ($\gamma_r = 11.4\gamma_{nr}$). Both resonators have been optimized to exhibit the same resonance wavelength $\lambda_R = 5.78\,\mu m$. Even if the critical coupling condition is equivalent to a maximum absorption condition, the electric field intensity can be higher in an over-coupled situation as it is the case here. Our objective is to reach the highest reflectivity difference, meaning a maximum absorption in the presence of the molecules which is not equivalent to critical coupling in their absence. Figures S2c-d show the evolution of the mean electric field intensity enhancement and the quality factor as a function of the coupling ratio γ_r/γ_{nr}. They show that it is possible to increase the bandwidth of operation (Q is decreasing) and at the same time increase the electric field enhancement. In practice, the slit dimensions are kept constant, while the period and the dielectric thickness are adjusted to get various coupling ratio at an identical resonant wavelength $\lambda_R = 5.78\,\mu m$.

1.4. SEIRA of PMMA in an 8 nm-thick slit resonator

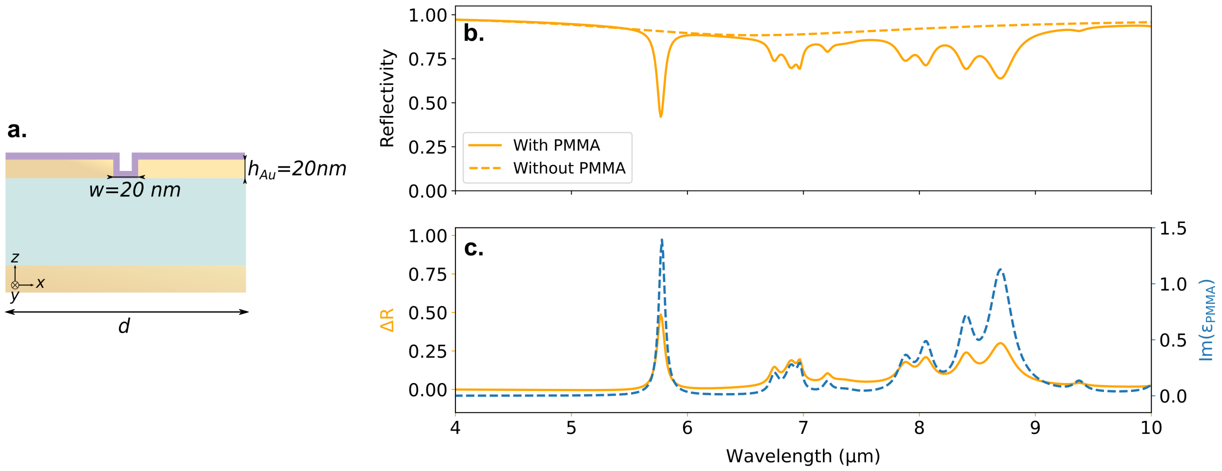

FIG. S3. **SEIRA of PMMA in a resonator with smaller slit.** **a** Scheme of the geometry. **b** Calculated spectra of a resonator with dimensions $h_{Au} = 20\,nm$, $h_{ZnS} = 530\,nm$, $w = 20\,nm$ and $d = 200\,nm$ covered with a continuous layer of PMMA of 8 nm. **c** Reflectivity difference ΔR=$R_{withoutPMMA}$-$R_{withPMMA}$ and imaginary part of the PMMA model.

A route to improve the sensitivity of the device is to reduce the volume of interaction.

Fig. S3 represents the theoretical response of a new design with a slit of section 20×20 nm resonating at 6.6 µm. Covered with an 8 nm thick layer of PMMA filling the slit and placed on top of the structure, a 0.48 reflectivity difference is obtained for the peak at 5.78 µm. This is a detection responsivity of 6%/nm, twice the result obtained experimentally, while maintaining the broad spectral enhancement. Nevertheless, reducing the slits to such small dimensions implies changing the fabrication process and the resist used for lithography.

2. DISCUSSION ON EXPERIMENTAL RESULTS AND COMPARISON TO SIMULATIONS

2.1. Deposition of PMMA

Experimental data of Fig. 4 were obtained by spin-coating various solutions of PMMA A2 diluted in anisole at different speed on identical resonators. Parameters are given in the Table S2. Ellipsometry measurements were performed next to the resonators to evaluate the deposited thickness. Table S4 displays the different deposition parameters of the PMMA layers.

TABLE S2. Table of deposited PMMA layers.

Dilution (Anisole:PMMA)	Spin-coat speed (rnds/min)	h_{PMMA} (nm)
2:1	2000	20.3
2:1	3000	16.9
2:1	4000	16.6
2:1	6000	14.7
3:1	2000	13.9
3:1	6000	12.8
4:1	6000	8.8

In order to monitor the homogeneity of the layers over the resonators, infrared spectra were acquired with a $50 \times 50\,\mu m^2$ window at different locations of the $1 \times 1 mm^2$ sample of the thinnest layer with a Vertex 70 coupled to an Hyperion 2000 microscope from Bruker.

Spectra are shown in Fig. S4a. They reveal slight variations of the spectra up to 5%. Atomic Force Microscopy (AFM) images were taken at different locations of the array with a Neaspec Nano-FTIR instrument to investigate the surface profile close to the resonators. The AFM tip used is a 20 nm silicon tip. The same measurements were performed on the sample with

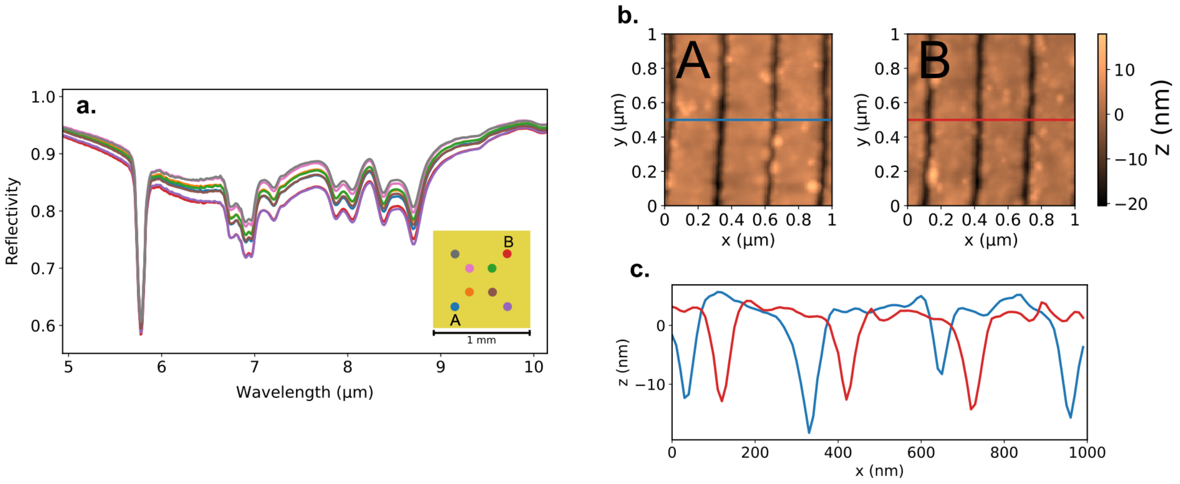

FIG. S4. **Homogeneity of the SEIRA response for an 8 nm-thick layer of PMMA a** Infrared spectra obtained on different locations of the array, represented in the inset. **b** AFM images correponding to points A and B of the inset. **c** Extracted profiles of AFM images. These results are obtained for the sample covered with the thinnest layer of PMMA.

the thickest layer of PMMA (20.3 nm) and are given in Fig. S5. In both cases, dips are visible at the slit positions and the surface is not flat. It must be emphasized that profiles extracted from those measurements show the convolution of the surface with the tip, which explains why it cannot reach the bottom of the slit. The surface on top of the ribbons is not completely smooth as PMMA could have accumulated on gold grains, which may influence the infrared response locally, as the resonators are very sensitive to the quantity of material inside the slit. Nevertheless, hundreds of resonators are measured for the acquisition of an infrared spectrum, the signal is averaged and corresponds to an effective thickness of PMMA.

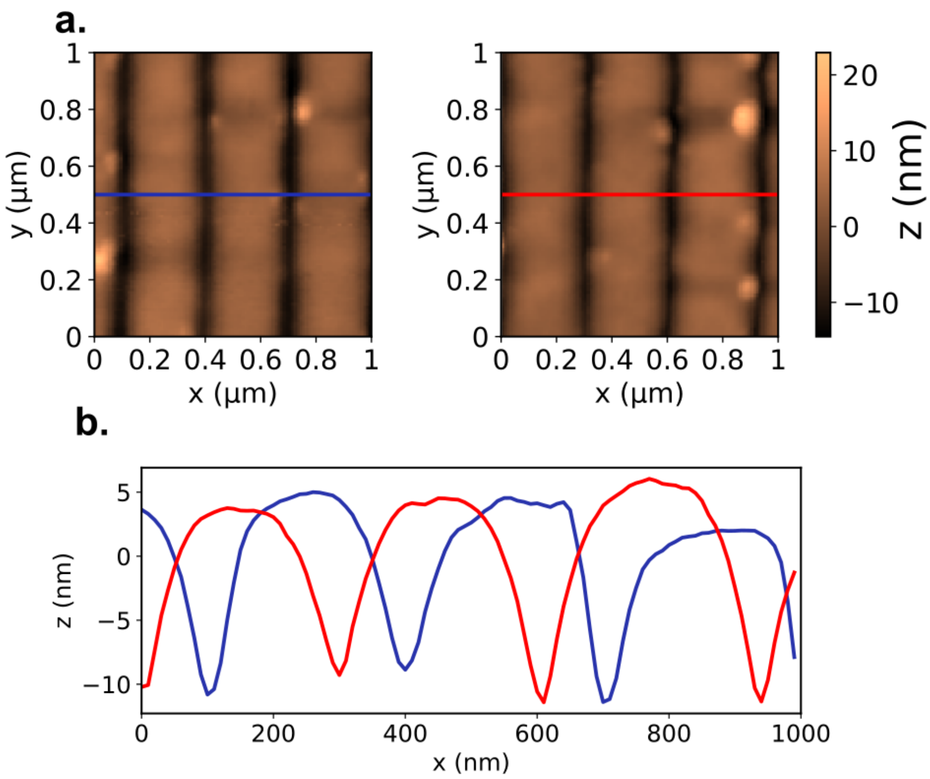

FIG. S5. **Homogeneity of the SEIRA response for a 20 nm-thick layer of PMMA a** AFM images correponding to points A and B of the inset. **b** Extracted profiles of AFM images. These results are obtained for the sample covered with the thickest layer of PMMA.

2.2. Deposition of DNT

Deposition of 2,4-dinitrotoluene was made thanks to a Microplotter II by Sonoplot. A glass micropipette of 20 μm diameter aperture tip is glued to a piezoelectric. The instrument enables to deposit very small volumes across a desired pattern with 10 μm lateral resolution. The glass pipette is put in contact with the surface and risen of 5 μm up so that when the pipette translate laterally, the solution is spread by capillarity. The volume, on the order of $2 \cdot 10^{-2}$ μL, is measured by the difference in the filling level of the capillary before and after deposition.

3. SEIRA ANALYTIC MODEL USING THE COUPLED-MODE THEORY

In this section, we use the temporal coupled mode theory (TCMT) to derive the analytical expression of the reflectivity from a resonator in presence of an absorber (represented by one absorption line). We end up with a very general statement: the optimal resonator for providing a large SEIRA signal over a large bandwidth (for spectrally separated absorption lines) is an over-coupled resonator whatever the molecules.

3.1. Temporal coupled mode theory (TCMT)

The TCMT describes the optical response of a resonator mode denoted a coupled to an absorber mode b via a coefficient denoted μ. The resonator frequency is ω_r and its decay rate is $\gamma_a = \gamma_r + \gamma_{nr}$. It is a sum of a radiative decay (which gives rise to the reflectivity) and a non-radiative one. The frequency resonance of mode b is ω_b and its decay rate γ_b is purely non radiative. The incident field s^+ is coupled to the resonator mode with a coefficient $\sqrt{2\gamma_r}$, which in turn emits into the reflected field s^-. This leads to the following set of equations:

$$\begin{cases} \dfrac{\mathrm{d}a}{\mathrm{d}t} = -i\omega_r a - (\gamma_r + \gamma_{nr})a + i\mu b + \sqrt{2\gamma_r}s^+ \\ \dfrac{\mathrm{d}b}{\mathrm{d}t} = -i\omega_b b - \gamma_b b + i\mu a \\ s^- = -s^+ + \sqrt{2\gamma_r}a. \end{cases} \tag{S3}$$

Next, we consider harmonic time variation: $a(t) = ae^{-i\omega t}$ and $b(t) = be^{-i\omega t}$, and insert the expression of b into the equation for a. Doing so, we obtain an equation for a only:

$$-i\omega a = -i(\omega_r + \omega_\mu)a - (\gamma_r + \gamma_{nr} + \gamma_\mu)a + \sqrt{2\gamma_r}s^+ \tag{S4}$$

where $\omega_\mu = \frac{\mu^2(\omega-\omega_b)}{(\omega-\omega_b)^2+\gamma_b^2}$ and $\gamma_\mu = \frac{\mu^2\gamma_b}{(\omega-\omega_b)^2+\gamma_b^2}$. Equation S4 reveals that the interaction with mode b can be taken into account adding a frequency varying shift ω_μ and a decay γ_μ to the resonator. Those terms drastically modify the reflectivity of the resonator close to the frequency resonance of the absorber leading to Eq. 2 of the main text. In Fig. S6, we plot $A(\tilde{\omega}, f)$ given by Eq. 2 of the main text, with $\tilde{\omega} = (\omega - \omega_r)/\gamma_{nr}$. We represent by a red dot the absorption of the bare resonator which is considered over-coupled and far detuned from the incident beam. The presence of the absorber shifts the position of the red dot in the diagram effectively changing the variables $\gamma_r/\gamma_{nr} \to \gamma_r/(\gamma_{nr} + \gamma_\mu)$ and

$(\omega - \omega_r)/\gamma_{nr} \to (\omega - \omega_r - \omega_\mu)/\gamma_{nr}$. This effect generally results in an increased reflectivity for an over-coupled resonator.

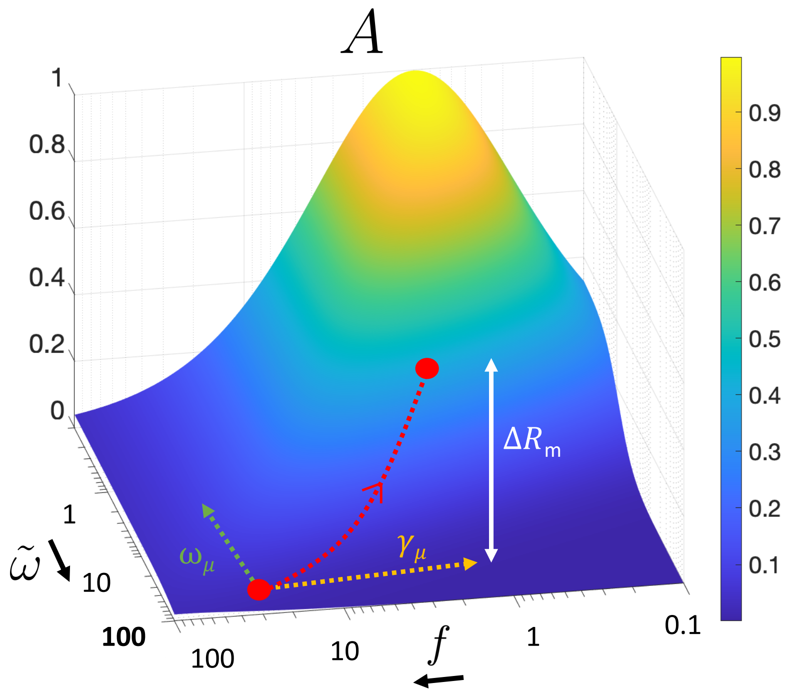

FIG. S6. **Absorption of the resonator in the TCMT model.** Absorption of the resonator as a function of $\tilde{\omega} = (\omega - \omega_r)/\gamma_{nr}$ and $f = \gamma_r/\gamma_{nr}$ computed from Eq. 2 of the main text. Without the absorber, the absorption is maximal when $\omega = \omega_r$ ($\tilde{\omega} = 0$) and $f = 1$ (critical coupling). The addition of an absorber into the resonator can be taken into account in this diagram changing the variables $\gamma_r/\gamma_{nr} \to \gamma_r/(\gamma_{nr}+\gamma_\mu)$ and $(\omega-\omega_r)/\gamma_{nr} \to (\omega-\omega_r-\omega_\mu)/\gamma_{nr}$. Since $\gamma_\mu > 0$, the operating point represented by a red dot always moves towards decreasing f.

To clarify this statement, we compute the reflectivity $r(\omega) = s^-/s^+$ from Eq. S3:

$$r(\omega) = \frac{(\omega_r - \omega)(\omega_b - \omega) + (\gamma_r - \gamma_{nr})\gamma_b - \mu^2 + i\left[(\omega_b - \omega)(\gamma_r - \gamma_{nr}) - (\omega_r - \omega)\gamma_b\right]}{(\omega - \omega_r)(\omega_b - \omega) + (\gamma_r + \gamma_{nr})\gamma_b + \mu^2 + i\left[(\omega_b - \omega)(\gamma_r + \gamma_{nr}) + (\omega_r - \omega)\gamma_b\right]}. \quad \text{(S5)}$$

Equation S5 is the exact solution of the TCMT and is equivalent to Eq. 2 of the main text. The reflectivity is a function of the frequency of the incident beam ω and depends on 6 physical parameters: ω_r, ω_b, γ_r,γ_{nr}, γ_b and μ.

TABLE S3. **Parameters of the TCMT model.** The parameters of the TCMT model for a resonator with the following dimensions: $h_{Au} = 45$ nm, $h_{ZnS} = 280$ nm, $w = 50$ nm, $d = 600$nm and a 45 nm thick PMMA layer filling the slits. The mode b corresponds to the absorption line of PMMA at 1730cm^{-1} (or 5.78μm). The parameters are given in rad.s^-1 and in units normalized by ω_r.

	ω_r	γ_r	γ_{nr}	ω_b	γ_b	μ
rad.s^-1	261×10^{12}	37.4×10^{12}	3.97×10^{12}	326×10^{12}	1.87×10^{12}	7.9×10^{12}
(...)/ω_r	1	0.14	0.015	1.25	0.0072	0.03

3.2. Typical values of the parameters of the TCMT model

In Table S3 we provide the parameters of the TCMT extracted from the reflectivity curve computed from one typical resolution of the Maxwell's equations. We observe some important features: the absorbing line is far detuned from the resonator $(\omega_b - \omega_r) \gg \gamma_b$, and the resonator is over-coupled: $\gamma_r \gg \gamma_{nr}$. In Fig. S7a we represent in solid blue a reflectivity curve computed from Eq. S5 using similar parameters than those presented in Table S3. To simplify the comparison between the different parameters, we normalize them by ω_r such that: $\omega_r = 1$, $\omega_b = 1.5$, $\gamma_r = 0.5$, $\gamma_{nr} = 0.01$, $\gamma_b = 0.01$ and $\mu = 0.03$. We observe a broad reflection dip (of typical width $\sim \gamma_r$) centered at the frequency resonance of the cavity ω_r together with a clearly visible narrow dip of typical width γ_b centered at the frequency resonance of the absorber ω_b.

3.3. SEIRA signal in the broadband regime

In this section, we analyse in detail Eq. S5 in order to demonstrate that over-coupled resonators are more sensitive to the presence of an absorbing line largely detuned from the resonator $\gamma_b \ll (\omega_b - \omega_r)$ than others. In this limit, that we denote the broadband regime, we can simplify the expression of $r(\omega)$ fixing $\omega = \omega_b$ in all terms of the form $(\omega - \omega_r)$ appearing in Eq. S5, and letting the frequency vary in terms like $(\omega - \omega_b)$. We obtain an expression of $r(\omega)$ valid in the vicinity of ω_b:

$$r(\omega) = \frac{(\omega_r - \omega_b)(\omega_b - \omega) + (\gamma_r - \gamma_{nr})\gamma_b - \mu^2 + i\left[(\omega_b - \omega)(\gamma_r - \gamma_{nr}) - (\omega_r - \omega_b)\gamma_b\right]}{(\omega_b - \omega_r)(\omega_b - \omega) + (\gamma_r + \gamma_{nr})\gamma_b + \mu^2 + i\left[(\omega_b - \omega)(\gamma_r + \gamma_{nr}) + (\omega_r - \omega_b)\gamma_b\right]}. \quad \text{(S6)}$$

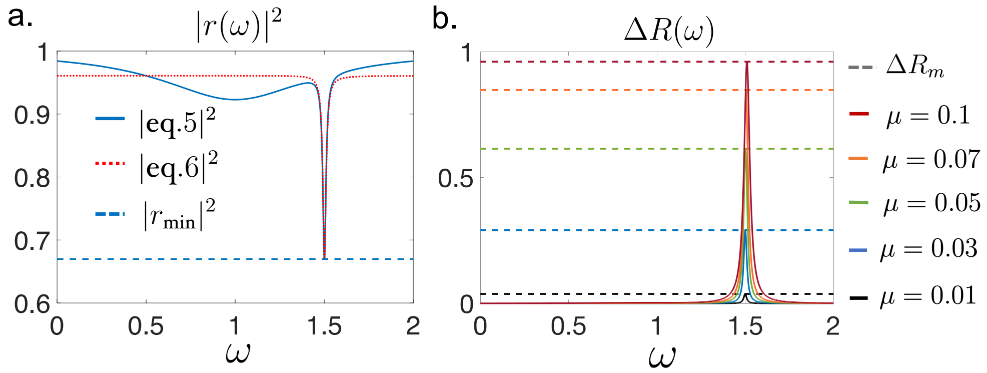

FIG. S7. **SEIRA signal spectra computed by the TCMT model.** **a** Reflectivity computed from the TCMT (blue solid) and its approximation in the vicinity of the absorber's resonance given by Eq. S6 (dotted red) as a function of the frequency of the incident light. In blue dashed line, we plot the minimum value of the reflectivity computed analytically. In **b** we plot $\Delta R(\omega) = |r_{\text{without}}(\omega)|^2 - |r_{\text{with}}(\omega)|^2$ for various coupling strength indicated by different colors and plot together its maximal value computed from Eq. S9. The parameters are given in units normalized by ω_r: $\omega_r = 1$, $\omega_b = 1.5$, $\gamma_r = 0.5$, $\gamma_{nr} = 0.01$, $\gamma_b = 0.01$ in (a,b). The coupling parameter $\mu = 0.03$ in **a** and varies from 0.01 to 0.1 in **b**.

In Fig. S7a we plot $|r(\omega)|^2$ given by Eq. S5 (solid blue) together with the expression given by Eq. S6 (dotted red). We observe that Eq. S6 perfectly captures the absorption peak due to the absorber and thus permits the analysis of the minimum of the reflectivity associated to it. To do so, we cast Eq. S6 in the standard form for a single resonance: $r(\omega) = P\frac{\omega-\omega_0}{\omega-\hat{\omega}}$ with :

$$\begin{cases} P = \dfrac{\omega_b - \omega_r - i(\gamma_r - \gamma_{nr})}{\omega_r - \omega_b - i(\gamma_r + \gamma_{nr})} \\ \text{Re}(\omega_0) = \omega_b + \dfrac{\mu^2(\omega_b - \omega_r)}{(\omega_b - \omega_r)^2 + (\gamma_r - \gamma_{nr})^2} \\ \text{Im}(\omega_0) = -\gamma_b + \dfrac{\mu^2(\gamma_r - \gamma_{nr})}{(\omega_b - \omega_r)^2 + (\gamma_r - \gamma_{nr})^2} \\ \text{Re}(\hat{\omega}) = \omega_b + \dfrac{\mu^2(\omega_b - \omega_r)}{(\omega_b - \omega_r)^2 + (\gamma_r + \gamma_{nr})^2} \\ \text{Im}(\hat{\omega}) = -\gamma_b - \dfrac{\mu^2(\gamma_r + \gamma_{nr})}{(\omega_b - \omega_r)^2 + (\gamma_r + \gamma_{nr})^2}. \end{cases} \tag{S7}$$

When the coupling constant μ is small enough, it can be shown that the minimum of reflectivity appears at the frequency $\omega \simeq \text{Re}(\hat{\omega}) = \omega_b + \frac{\mu^2(\omega_b-\omega_r)}{(\omega_b-\omega_r)^2+(\gamma_r+\gamma_{nr})^2}$ and equals $r_{min} =$

$P\frac{\text{Im}(\omega_0)}{\text{Im}(\hat{\omega})}$. In Fig. S7a, we plot $|r_{min}|^2$ in horizontal dashed blue line and show good agreement with eqs. S5 and S6.

As a figure of merit for quantifying the SEIRA signal, we study the maximum value of $\Delta R(\omega) = |r_{\text{without}}(\omega)|^2 - |r_{\text{with}}(\omega)|^2$ that we denote ΔR_m. Using $r_{min} = P\frac{\text{Im}(\omega_0)}{\text{Im}(\hat{\omega})}$, we can write:

$$\Delta R_m = |P|^2 \left(1 - \frac{\text{Im}(\omega_0)^2}{\text{Im}(\hat{\omega})^2}\right). \tag{S8}$$

Next, we insert the expressions of P, $\text{Im}(\omega_0)$ and $\text{Im}(\hat{\omega})$ provided in Eq. S7 and end up with an expression of ΔR_m that depends on 3 normalized parameters $\tilde{\mu} = \mu^2/(\gamma_b\gamma_{nr})$, $f = \gamma_r/\gamma_{nr}$ and $\tilde{\Delta} = (\omega_b - \omega_r)/\gamma_{nr}$:

$$\Delta R_m = \frac{\tilde{\Delta}^2 + (f-1)^2}{\tilde{\Delta}^2 + (f+1)^2} \left[1 - \frac{\left(1 - \frac{\tilde{\mu}(f-1)}{\tilde{\Delta}^2+(f-1)^2}\right)^2}{\left(1 + \frac{\tilde{\mu}(f+1)}{\tilde{\Delta}^2+(f+1)^2}\right)^2}\right]. \tag{S9}$$

In Fig. S7b we plot in dashed horizontal lines the value of ΔR_m computed from Eq. S9 for different values of the coupling parameter μ. Doing so, we confirm that it properly captures the maximum value of ΔR as a function of ω.

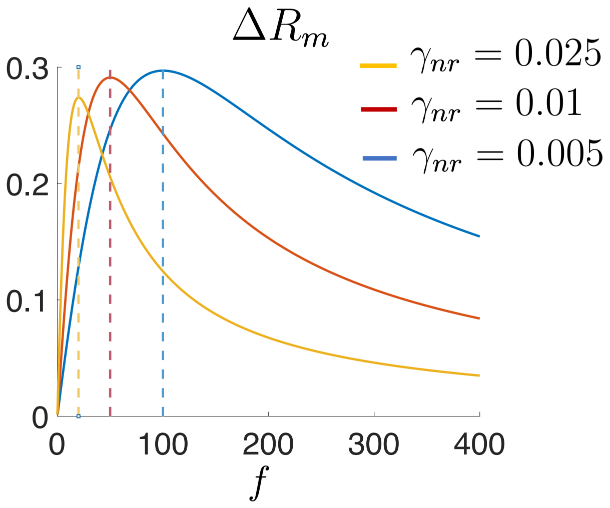

FIG. S8. **SEIRA signal as a function of the coupling ratio in the TCMT model.** ΔR_m as a function $f = \gamma_r/\gamma_{nr}$. To vary f, we fix γ_{nr} and let γ_r vary. In dashed, we plot the value $f = \tilde{\Delta} = (\omega_b - \omega_r)/\gamma_{nr}$ associated to each value of γ_{nr} to highlight the position of the maximum of $\Delta R_m(f)$. The normalized parameters are : $\omega_r = 1$, $\omega_b = 1.5$, $\gamma_r = 0.5$, $\gamma_b = 0.01$ and $\mu = 0.03$. We consider various values of γ_{nr} as indicated by different colors.

3.4. Sensitivity of the SEIRA signal in the broadband regime

In this subsection, we use Eq. S9 in order to study the sensitivity of the SEIRA signal to a small quantity of absorber. In the TCMT model, the quantity of absorber is proportional to μ^2. We, thus expand ΔR_m in powers of μ^2 close to $\mu^2 = 0$ and obtain:

$$\Delta R_m \simeq \frac{4}{\gamma_b \gamma_{nr}} \underbrace{\frac{\left(-f + \tilde{\Delta}^2 f + f^3\right)}{\left(1 + \tilde{\Delta}^2 + 2f + f^2\right)^2}}_{S(f)} \times \mu^2 + O(\mu^4). \tag{S10}$$

Equation S10 tells us that a small quantity of absorber induces a change in the SEIRA signal proportional to the function $S(f)$. In the limit of an absorbing line far detuned from the resonator ($\tilde{\Delta} \gg 1$), we can show that $S(f)$ is maximal for $f_{\max} = \tilde{\Delta} \gg 1$, deep inside the over-coupled regime. This is the main result of the section. It means that the most sensitive resonator to a small quantity of absorber far detuned from the resonator is over-coupled. In Fig. S8 we support this claim, plotting $\Delta R_m(f)$ for sufficiently small μ such that the development given in Eq. S10 remains valid. To do so, we use as normalized parameters : $\omega_r = 1$, $\omega_b = 1.5$, $\gamma_r = 0.5$, $\gamma_b = 0.01$ and $\mu = 0.03$ and we consider various values of γ_{nr} as indicated by different colors. We clearly observe the existence of a maximum of the SEIRA signal for $f_{\max} = \tilde{\Delta} \gg 1$ (represented in dashed vertical lines of different colors) deep inside the over-coupled regime.

Eventually, we can inject the optimal condition on $f = \tilde{\Delta} \gg 1$ into Eq. S9 and obtain a simplified expression of ΔR_m:

$$\Delta R_m \simeq \left[1 - \frac{\left(1 - \frac{\tilde{\mu}}{2\tilde{\Delta}}\right)^2}{\left(1 + \frac{\tilde{\mu}}{2\tilde{\Delta}}\right)^2}\right] \tag{S11}$$

which depends on a single parameter $X = \frac{\tilde{\mu}}{2\tilde{\Delta}}$. Since $f(X) = 1 - (1 - X)^2/(1 + X)^2$ is maximum and equals to 1 when $X = 1$, we demonstrate that ΔR_m can be as large as 1 when $\mu = \sqrt{2(\omega_b - \omega_r)\gamma_b}$.

3.5. Critical coupling mediated by the absorber

Equation S11 was obtained pushing both f and $\tilde{\Delta}$ towards ∞. Here, we discuss the condition to maximize the SEIRA signal ΔR_m using a more general method that do not

need assumptions on the physical parameters. To do so, we go back to Eq. S4 and solve the critical coupling condition mediated by mode b. It writes:

$$\begin{cases} \omega = \omega_\mu + \omega_r, \\ \gamma_r = \gamma_{nr} + \gamma_\mu \end{cases} \tag{S12}$$

with $\omega_\mu = \frac{\mu^2(\omega-\omega_b)}{(\omega-\omega_b)^2+\gamma_b^2}$ and $\gamma_\mu = \frac{\mu^2\gamma_b}{(\omega-\omega_b)^2+\gamma_b^2}$. Equation S4 tells us that the reflectivity in presence of the absorber reaches unity when $\omega = \omega_c$ and $\mu = \mu_c$ with

$$\begin{cases} \omega_c = \dfrac{\omega_r\gamma_b - \omega_b(\gamma_r - \gamma_{nr})}{\gamma_b + \gamma_{nr} - \gamma_r} \\ \mu_c^2 = \dfrac{(\omega_c - \omega_b)^2 + \gamma_b^2}{\gamma_b}(\gamma_r - \gamma_{nr}). \end{cases} \tag{S13}$$

We note that Eq. S13 is equivalent to the condition obtained using Eq. S11: $\mu = \sqrt{2(\omega_b - \omega_r)\gamma_b}$ in the limit $(\gamma_b, \gamma_{nr}) \ll \gamma_r$. The expression of the SEIRA signal under the constraints of Eq. S13 is $\Delta R_m = |P|^2$. For instance, using the parameters of Fig. S7b, we find $\omega_c = 1.51$, $\mu_c = 0.1$ and $|P|^2 = 0.96$ in perfect agreement with the numerical observation.

4. LINEAR BEHAVIOR OF THE SEIRA SIGNAL THANKS TO AN HOMOGRAPHIC APPROXIMATION

We have seen on Fig. 4 that the reflectivity is a non-linear function of the thickness. Here, we show that using Eq. 1, derived from the temporal coupled mode theory[S8, S9], and a linear model of its parameters as a function of the thickness, we obtain an accurate description of the resonator.

4.1. Homographic approximation in the temporal coupled-mode theory

The absorption of the resonator can thus be written as:

$$A = \frac{4\gamma_r\gamma_{nr}}{(\omega - \omega_r)^2 + (\gamma_r + \gamma_{nr})^2}, \tag{S14}$$

with γ_r the radiative damping rate, γ_{nr} the non radiative damping rate, ω the frequency and ω_r the resonance frequency. To investigate the reflectivity amplitude r behavior in the vicinity of a pole $\hat{\omega} = \hat{\omega}' + i\hat{\omega}''$ ($\hat{\omega}'' < 0$ due to the $\exp(-i\omega t)$ convention taken for the

dependence of the electromagnetic field), it can be expressed thanks to a Laurent series accounting only for this pole [S10]:

$$r(\omega) \simeq a + \frac{b}{\omega - \hat{\omega}} \tag{S15}$$

where a and b are unknown parameters. This reflectivity amplitude can be written as an homographic function:

$$r(\omega) \simeq a\frac{\omega - \omega_0}{\omega - \hat{\omega}} \tag{S16}$$

with $\omega_0 = \hat{\omega} - \frac{b}{a} = \omega_0' + i\omega_0''$. In our system, since there is no transmission, the absorption is linked to the reflection R and its amplitude r by $A = 1 - R = 1 - |r|^2$. Rigorously, the Laurent series approximation is only valid near the pole, but we have verified numerically that is was still valid for our structure when $\omega \to 0$. In that case, the reflectivity amplitude of the resonator converges towards the reflectivity of a gold surface and thus $|a|^2 \simeq 1$. The reflectivity is then given by the equation:

$$R = |r(\omega)|^2 = \frac{(\omega - \omega_0')^2 + \omega_0''^2}{(\omega - \hat{\omega}')^2 + \hat{\omega}''^2}$$

Thanks to Eqs. S14-S16, it is possible to identify the losses of the resonator as a function of ω_0 and $\hat{\omega}$ when considering that $\omega_0' \simeq \hat{\omega}'$ (which is verified numerically):

$$\begin{cases} 4\gamma_r\gamma_{nr} = \hat{\omega}''^2 - \omega_0''^2 \\ \gamma_r + \gamma_{nr} = -\hat{\omega}'' \end{cases} \tag{S17}$$

We take $-\hat{\omega}''$ since γ_r and γ_{nr} are both positive values. Besides, γ_r and γ_{nr} are interchangeable and are both the solutions of a second degree equation. The coupling ratio of the resonator is defined as $\frac{\gamma_r}{\gamma_{nr}}$, and is greater than one for over-coupled configurations of the resonator. In that case, the radiative and non radiative damping rates are given by the following equations:

$$\begin{cases} \gamma_r = \frac{-\hat{\omega}'' + |\omega_0''|}{2} \\ \gamma_{nr} = \frac{-\hat{\omega}'' - |\omega_0''|}{2} \end{cases} \tag{S18}$$

Their values are exchanged for an undercoupled resonator ($\frac{\gamma_r}{\gamma_{nr}} < 1$) and equalled at critical coupling ($\gamma_r = \gamma_{nr}$).

4.2. Linearization of the homographic approximation

We introduce a linearization of the three parameters involved in Eq. S16 as a function of $x = (\epsilon_{\text{PMMA}} - 1)\frac{h_{\text{PMMA}}}{h_{\text{Au}}}$:

$$\begin{cases} a(x) = a^0 + a^1 x \\ \hat{\omega}(x) = \hat{\omega}^0 + \hat{\omega}^1 x \\ \omega_0(x) = \omega_0^0 + \omega_0^1 x \end{cases} \tag{S19}$$

The complex parameters a^0, $\hat{\omega}^0$ and ω_0^0 are usually determined thanks to electromagnetic computations at three wavelengths. Similarly, three electromagnetic computations can be done with the slit filled with PMMA (*i.e.,* $h_{\text{PMMA}} = h_{\text{Au}}$) to determine the three additional parameters of the linearized model a^1, $\hat{\omega}^1$ and ω_0^1. Then, it is possible to write the reflectivity amplitude using a first order expansion when x is small:

$$r(x) = r(0) + Cx + o(x), \tag{S20}$$

where C can be determined from straighforward algebra as a function of the 6 parameters introduced in Eq. S19. This means that the difference $\Delta R = |r(x)|^2 - |r(0)|^2$ can be written as:

$$\Delta R \simeq 2\frac{h_{\text{PMMA}}}{h_{\text{Au}}}\Re(r(0)^*\, C(\epsilon_{\text{PMMA}} - 1)) \tag{S21}$$

This analysis highlights the linear dependence to the amount of molecules of the reflectivity difference when x is small. The validity of this linear dependence is limited by the strength of the absorption, which explains the difference of behaviors seen in Fig. **??**. It also enables the computation of $\Delta R(x, \lambda)$ from six complex constants for any molecule with $\epsilon(\lambda)$ known.

4.3. Comparison of the analytic model to electromagnetic simulations

Two examples are given in the following figure S9 where the reflectivity difference spectra are plotted as function of the thickness of the analyte's layer in the slit of the resonator and on top of the gold ribbons for DNT and PMMA. In comparison, the spectra obtained from RCWA calculations are shown in the right panels. It is seen that the agreement is excellent for all parameter values.

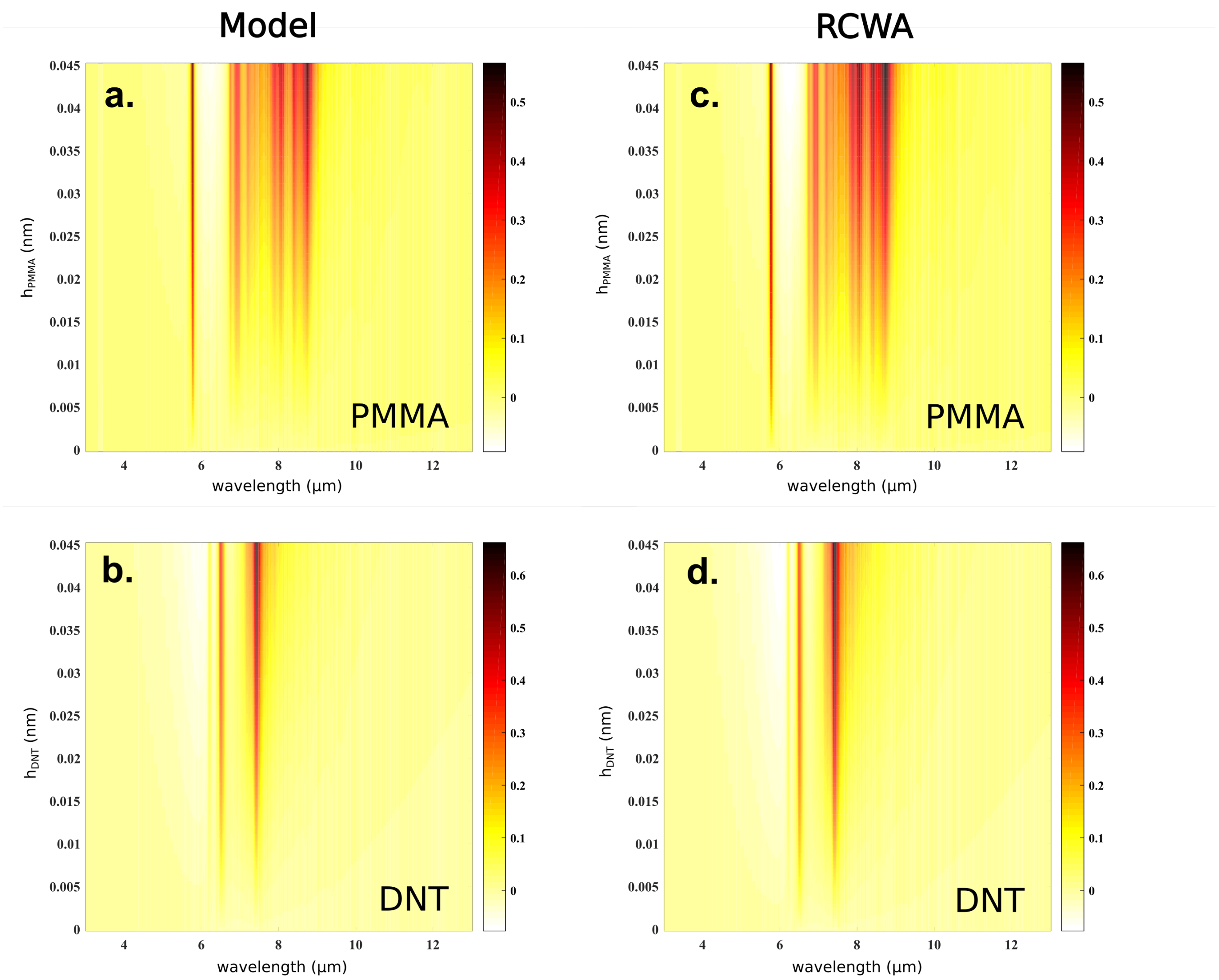

FIG. S9. **Comparison of the linearized model with electromagnetic computations for PMMA and DNT. a** Evolution of the reflectivity difference ΔR as a function of wavelength and of the analyte layer thickness in the slit and on top of a resonator (dimensions h_{Au}=45 nm, h_{ZnS}=280 nm, w=50 nm and d=600 nm) for **a** PMMA and **b** DNT calculated with the linearized model. The exact electromagnetic computations with RCWA are given for **c** PMMA and d) DNT.

5. COMPARISON TO EXPERIMENTAL SEIRA OF PMMA

Table S4 summarizes the results obtained in the literature for experimental SEIRA for PMMA.

TABLE S4. **Table of experimental SEIRA signals obtained with PMMA in literature.**

Structure	PMMA Thickness	Infrared signal	Targeted mode	Article
Au asymmetric SRR	100 nm	ΔR = 15 %	1730 cm^{-1}	Lahiri *et al.* (2009) [S11]
Dual band Au asymmetric crossed nanoantenna	4 nm	ΔR = 10 % ΔR = 2 %	1730 cm^{-1} 2960-2990 cm^{-1}	Chen *et al.* (2012) [S12]
Dipolar rod antenna	8 nm	ΔR = 25 %	1730 cm^{-1}	Adato *et al.* (2013) [S9]
Au bowtie arrays	31-41 nm	ΔR = 2-3 %	1730 cm^{-1}	Hoffman *et al.* (2013) [S13]
Au gratings	< 30 nm	ΔR = 6 %	1730 cm^{-1}	Wang *et al.* (2013) [S14]
InAs nanopillar arrays	50 nm	ΔR = 1 %	752 and 842 cm^{-1}	Law *et al.* (2013) [S15]
ITO nanoantennas	50 nm	ΔT = 8 %	1730 cm^{-1}	Abb *et al.* (2014) [S16]
Ag coated diffraction gratings	140 nm	ΔR = 20 %	2950-2994 cm^{-1}	Petefish *et al.* (2014) [S17]
Au dolmen type slit structure	12.8 nm	ΔR = 9 %	1730 cm^{-1}	Cheng *et al.* (2015) [S18]
Concentric Au rings	50 nm	ΔR = Few %	1100-1300 and 1730 cm^{-1}	Dayal *et al.* (2016) [S19]
Dual resonant Au stripes	10 nm	ΔR = 20 % ΔR = 2 %	1730 cm^{-1} 2960-2990 cm^{-1}	Cetin *et al.* (2016) [S20]
GZO bowtie arrays	50 nm	ΔT = 2-3 %	1100-1300 and 1730 cm^{-1}	Habeeb *et al.* (2016) [S21]
InAsSb nanogratings	200 nm	ΔR = 1 %	990 cm^{-1}	Barho *et al.* (2016) [S22]
Au nanorod arrays	40 nm	ΔR = 10 % ΔR < 2 %	1730 cm^{-1} 1200 - 1485 cm^{-1}	Braun *et al.* (2016) [S23]
Asymmetrical Au nanoantenna	50 nm	ΔT = 20 %	1730 cm^{-1}	Ishikawa *et al.* (2017) [S24]
Au nanotriangles	_	ΔT = 6 %	1730 cm^{-1}	Chen *et al.* (2017) [S25]
Au fan pad rods with 30 nm gap	50 nm	ΔR = 14.5 ΔR < 2 % %	1730 cm^{-1} 2955 and 2989 cm^{-1}	Yue *et al.* (2019) [S26]
Disordered slit antenna	30 nm	ΔR = 5 %	1730 cm^{-1}	Armelles *et al.* (2022) [S27]
Helmholtz optical resonator	8.8 nm	ΔR = 27 % ΔR = 3-25 %	1730 cm^{-1} and 1066 - 1700 cm^{-1}	This work

REFERENCES

[S1] Tsuda, S., Yamaguchi, S., Kanamori, Y. & Yugami, H. Spectral and angular shaping of infrared radiation in a polymer resonator with molecular vibrational modes. *Optics Express* **26**, 6899 (2018).

[S2] Bouchon, P., Pardo, F., Haïdar, R. & Pelouard, J.-L. Fast modal method for subwavelength gratings based on B-spline formulation. *Journal of the Optical Society of America A* **27**, 696 (2010).

[S3] Hugonin, J.-P. & Lalanne, P. Reticolo software for grating analysis. *Institut d'Optique, Palaiseau, France* (2005).

[S4] Klein, C. A. Room-temperature dispersion equations for cubic zinc sulfide. *Applied Optics* **25**, 1873 (1986).

[S5] Rakić, A. D., Djurišić, A. B., Elazar, J. M. & Majewski, M. L. Optical properties of metallic films for vertical-cavity optoelectronic devices. *Appl. Opt.* **37**, 5271–5283 (1998).

[S6] Palik, E. D. *Handbook of Optical Constants of Solids.* Academic Press (1985).

[S7] Chevalier, P., Bouchon, P., Haïdar, R. & Pardo, F. Optical Helmholtz resonators. *Appl. Phys. Lett.* **105**, 071110 (2014).

[S8] Fan, S., Suh, W. & Joannopoulos, J. D. Temporal coupled-mode theory for the fano resonance in optical resonators. *Journal of the Optical Society of America A* **20**, 569 (2003).

[S9] Adato, R., Artar, A., Erramilli, S. & Altug, H. Engineered Absorption Enhancement and Induced Transparency in Coupled Molecular and Plasmonic Resonator Systems. *Nano Letters* **13**, 2584–2591 (2013).

[S10] Enoch, S. & Bonod, N. (eds.) *Plasmonics: From Basics to Advanced Topics*, vol. 167 of *Springer Series in Optical Sciences* (Springer Berlin Heidelberg, 2012).

[S11] Lahiri, B., Khokhar, A. Z., De La Rue, R. M., McMeekin, S. G. & Johnson, N. P. Asymmetric split ring resonators for optical sensing of organic materials. *Optics Express* **17**, 1107 (2009).

[S12] Chen, K., Adato, R. & Altug, H. Dual-Band Perfect Absorber for Multispectral Plasmon-Enhanced Infrared Spectroscopy. *ACS Nano* **6**, 7998–8006 (2012).

[S13] Hoffmann, J. M. et al. Low-Cost Infrared Resonant Structures for Surface-Enhanced Infrared Absorption Spectroscopy in the Fingerprint Region from 3 to 13 m. *The Journal of Physical Chemistry C* **117**, 11311–11316 (2013).

[S14] Wang, T., Nguyen, V. H., Buchenauer, A., Schnakenberg, U. & Taubner, T. Surface enhanced infrared spectroscopy with gold strip gratings. *Optics Express* **21**, 9005 (2013).

[S15] Law, S., Yu, L., Rosenberg, A. & Wasserman, D. All-semiconductor plasmonic nanoantennas for infrared sensing. *Nano Letters* **13**, 4569–4574 (2013).

[S16] Abb, M., Wang, Y., Papasimakis, N., de Groot, C. H. & Muskens, O. L. Surface-Enhanced Infrared Spectroscopy Using Metal Oxide Plasmonic Antenna Arrays. *Nano Letters* **14**, 346–352 (2014).

[S17] Petefish, J. W. & Hillier, A. C. Angle-Tunable Enhanced Infrared Reflection Absorption Spectroscopy via Grating-Coupled Surface Plasmon Resonance. *Analytical Chemistry* **86**, 2610–2617 (2014).

[S18] Cheng, F., Yang, X. & Gao, J. Ultrasensitive detection and characterization of molecules with infrared plasmonic metamaterials. *Scientific Reports* **5**, 14327 (2015).

[S19] Dayal, G., Chin, X. Y., Soci, C. & Singh, R. High- Q Plasmonic Fano Resonance for Multiband Surface-Enhanced Infrared Absorption of Molecular Vibrational Sensing. *Advanced Optical Materials* **5**, 1600559 (2017).

[S20] Cetin, A. E. et al. Quantification of Multiple Molecular Fingerprints by Dual-Resonant Perfect Absorber. *Advanced Optical Materials* **4**, 1274–1280 (2016).

[S21] Habeeb, A. A. et al. Surface plasmonic resonances and enhanced IR spectra in GZO nanotriangle arrays. *Materials Letters* **172**, 36–39 (2016).

[S22] Barho, F. B. et al. All-semiconductor plasmonic gratings for biosensing applications in the mid-infrared spectral range. *Optics Express* **24**, 16175 (2016).

[S23] Braun, A. & Maier, S. A. Versatile direct laser writing lithography technique for surface enhanced infrared spectroscopy sensors. *ACS Sensors* **1**, 1155–1162 (2016).

[S24] Ishikawa, A., Hara, S., Tanaka, T., Zhang, X. & Tsuruta, K. Robust plasmonic hot-spots in a metamaterial lattice for enhanced sensitivity of infrared molecular detection. *Applied Physics Letters* **111**, 243106 (2017).

[S25] Chen, K., Duy Dao, T. & Nagao, T. Tunable Nanoantennas for Surface Enhanced Infrared Absorption Spectroscopy by Colloidal Lithography and Post-Fabrication Etching. *Scientific*

Reports **7**, 44069 (2017).

[S26] Yue, W. et al. Multiple-resonant pad-rod nanoantennas for surface-enhanced infrared absorption spectroscopy. *Nanotechnology* **30**, 465206 (2019).

[S27] Armelles, G., Cebollada, A., Cava, D. G., Alvarez-Malmagro, J. & Vélez, M. Surface Enhanced Infrared Characterization Using Disordered Slit-Antenna Arrays for the Detection of Electrodeposited Cytochrome C. *Plasmonics* (2021).
